# Supplementary figures and images for: Assessing a behavioral nudge on healthcare leaders’ intentions to implement evidence-based practices
Source: PLoS One. 2024 Nov 22;19(11):e0311442. doi: 10.1371/journal.pone.0311442 (PMC11584086; doi:10.1371/journal.pone.0311442)

**S1 File. Survey peer comparison report**


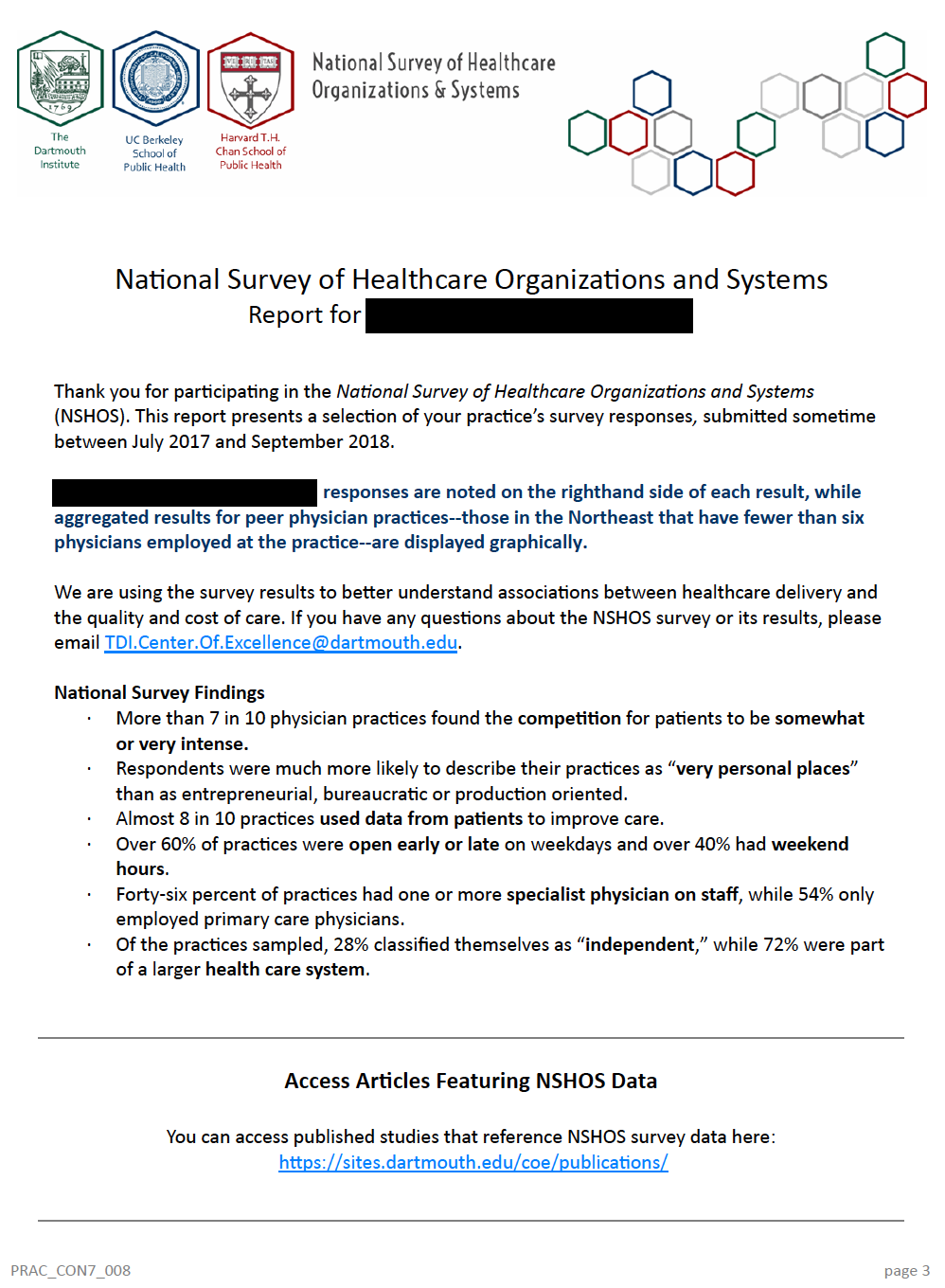


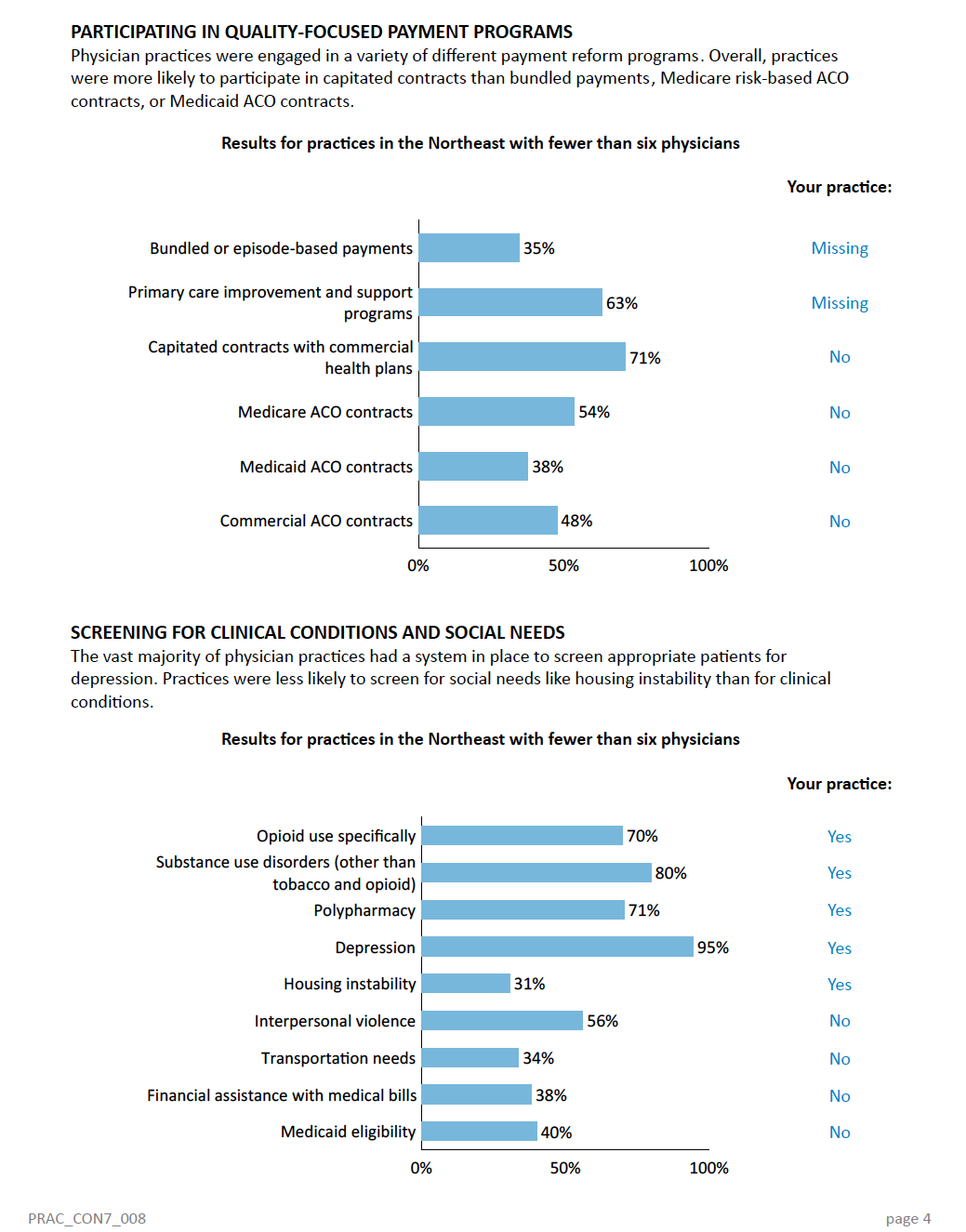


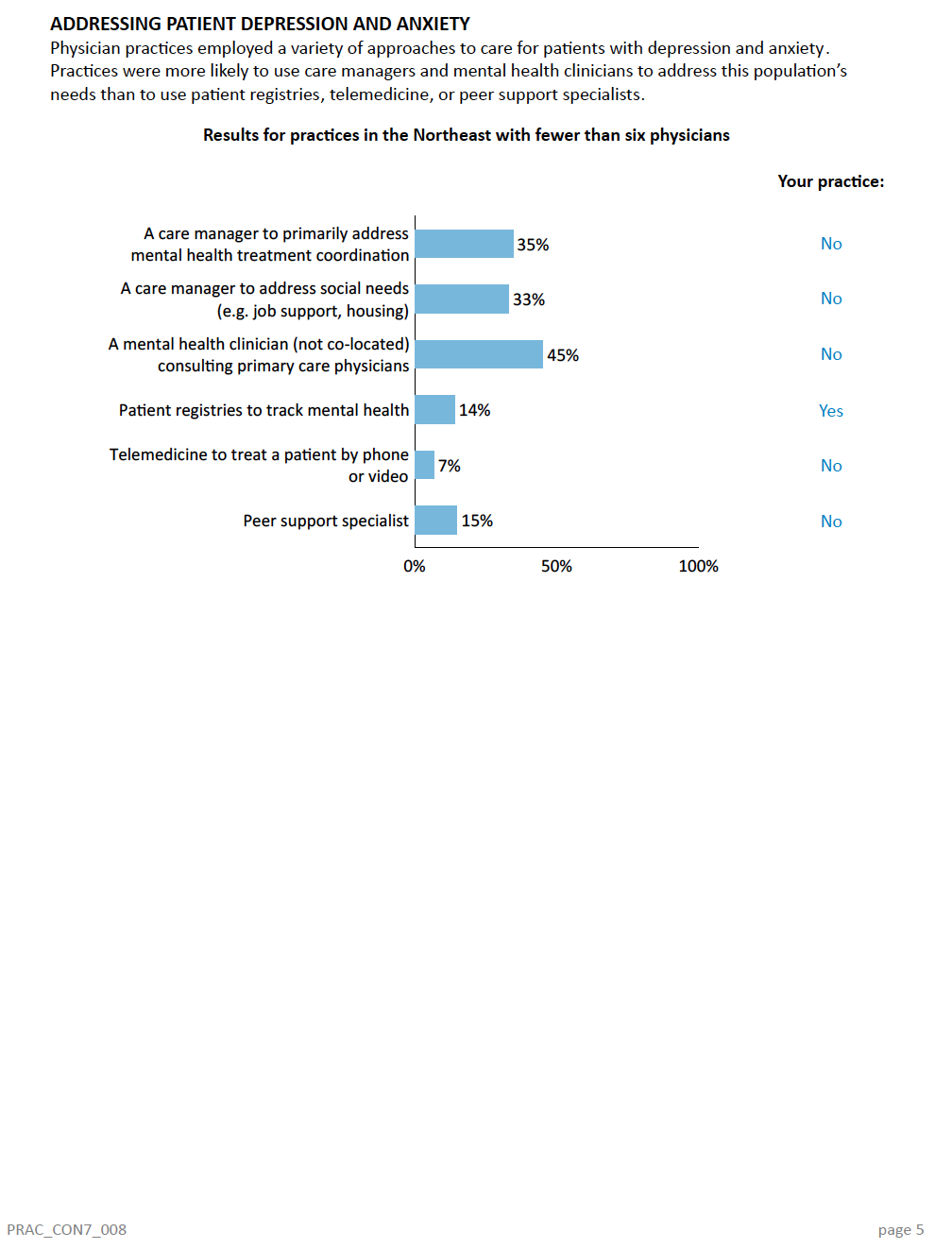


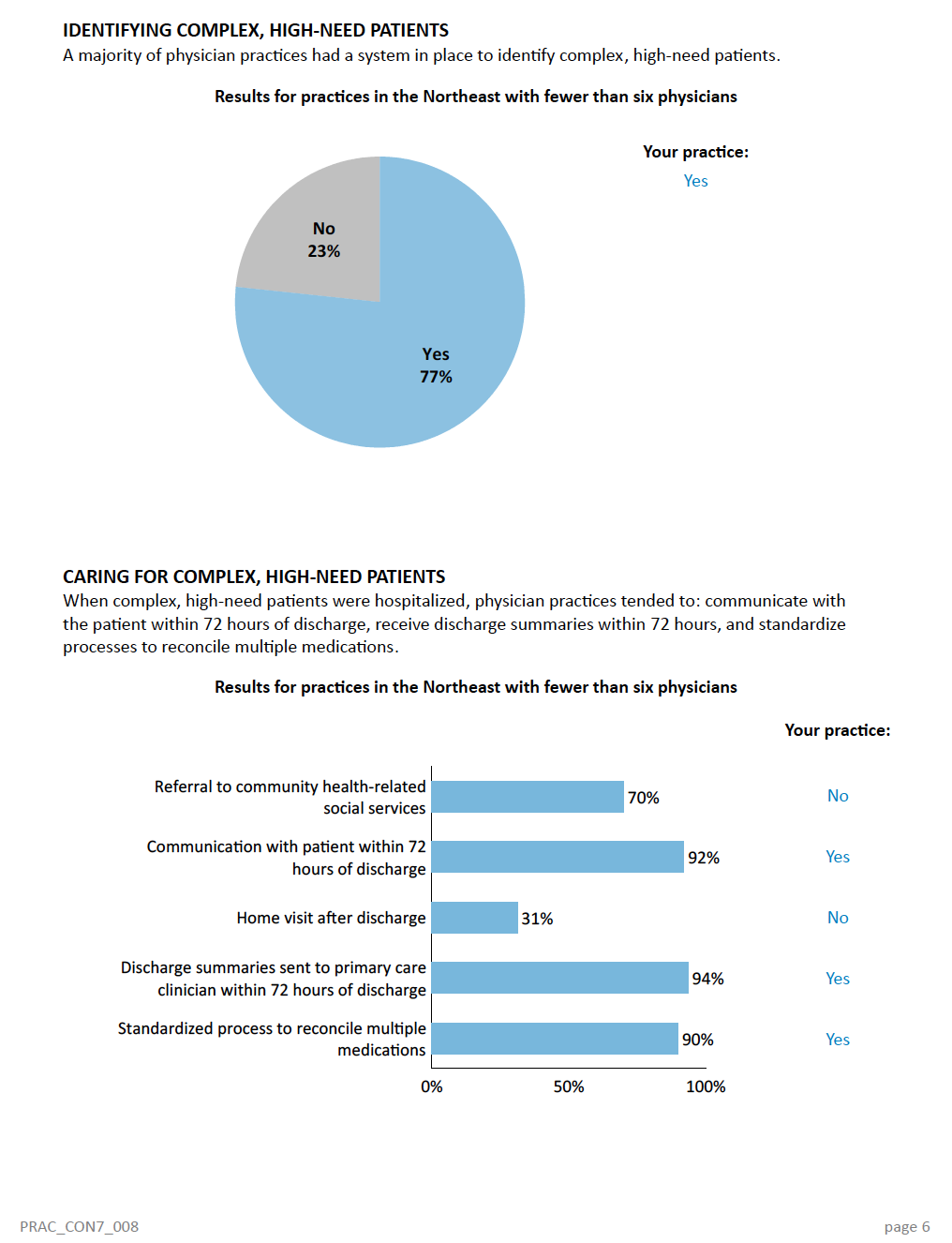


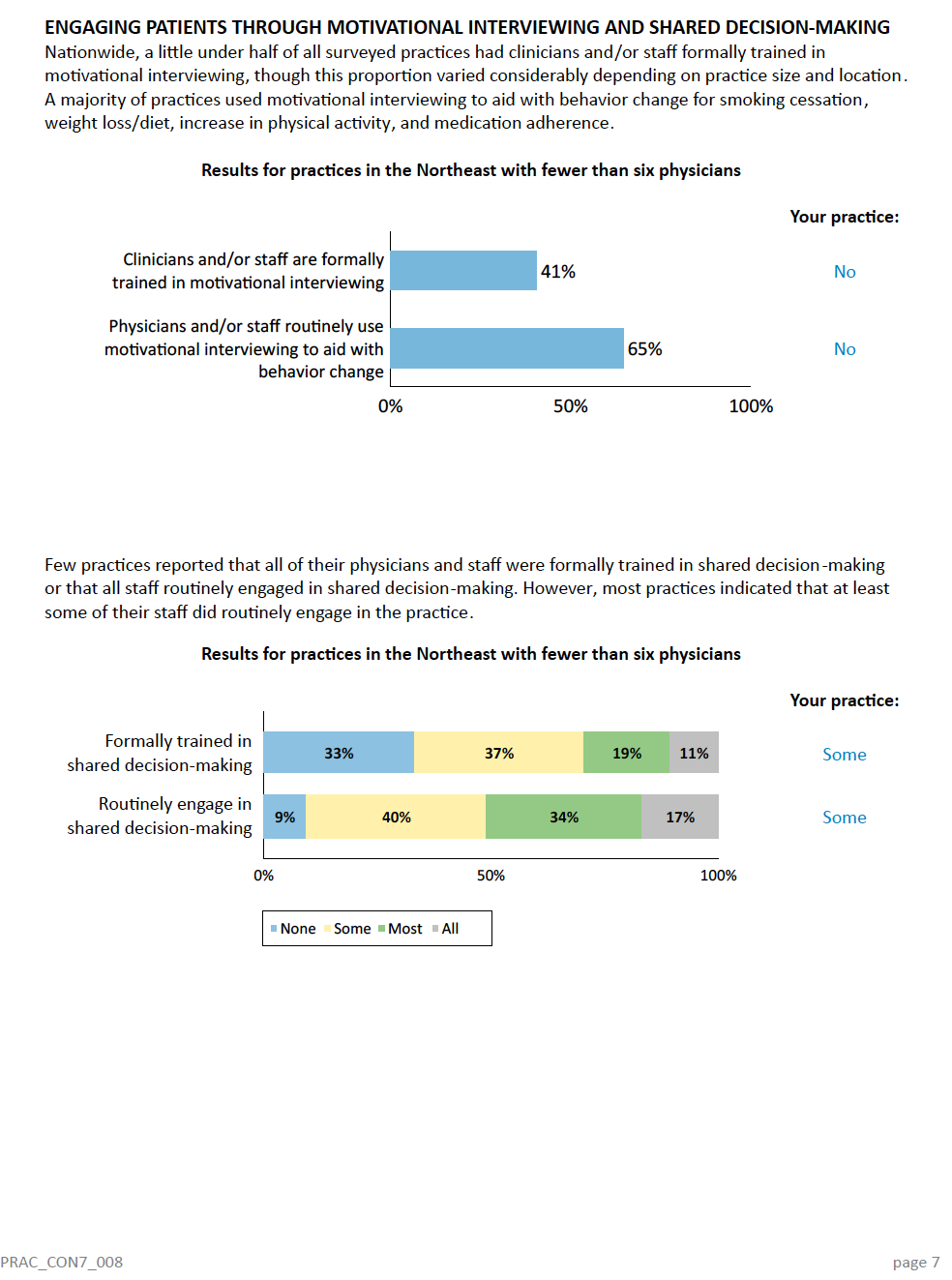


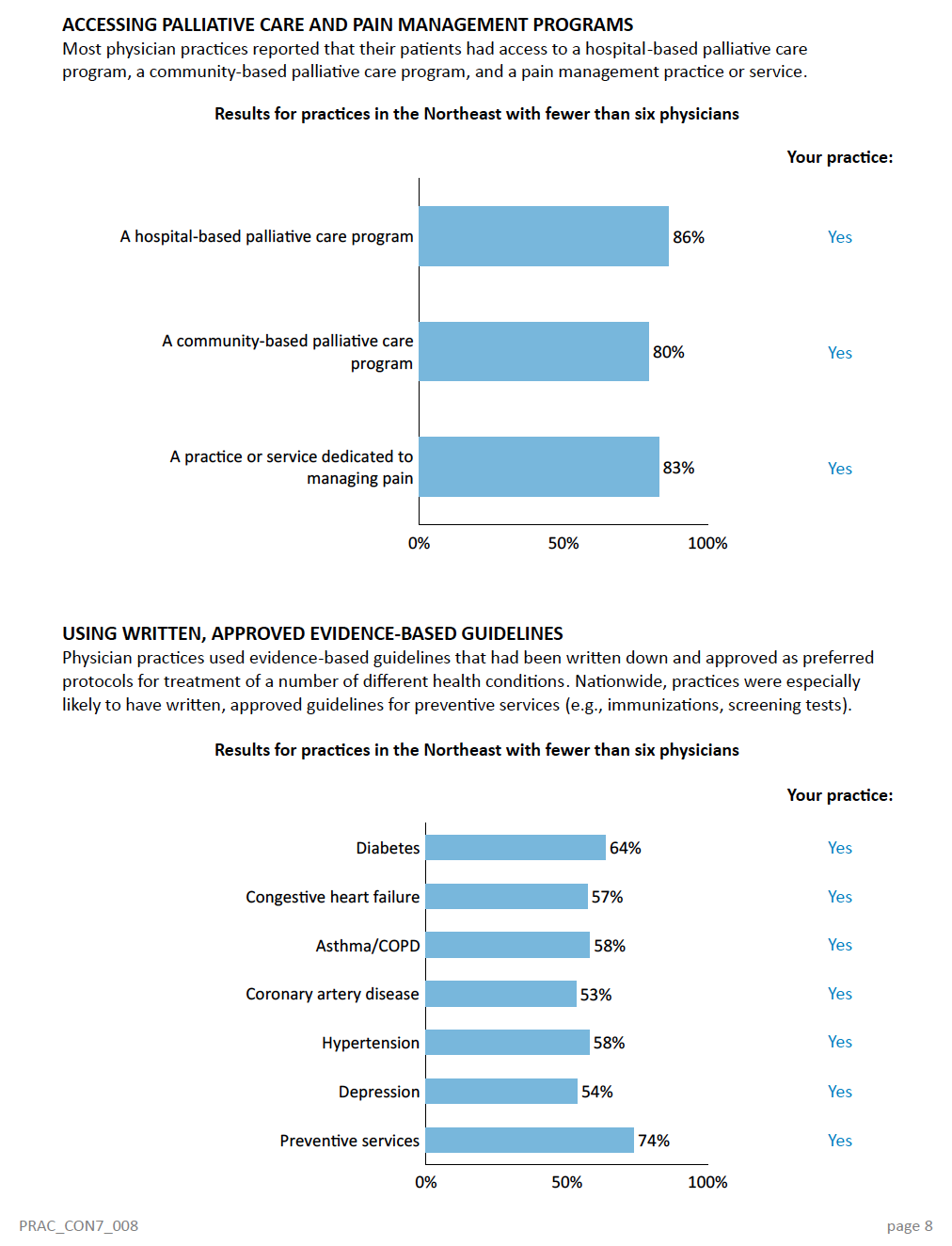

Supplement: S1 File — (DOCX) [file pone.0311442.s001.docx]

**S3 File. Nudge hard copy letter**


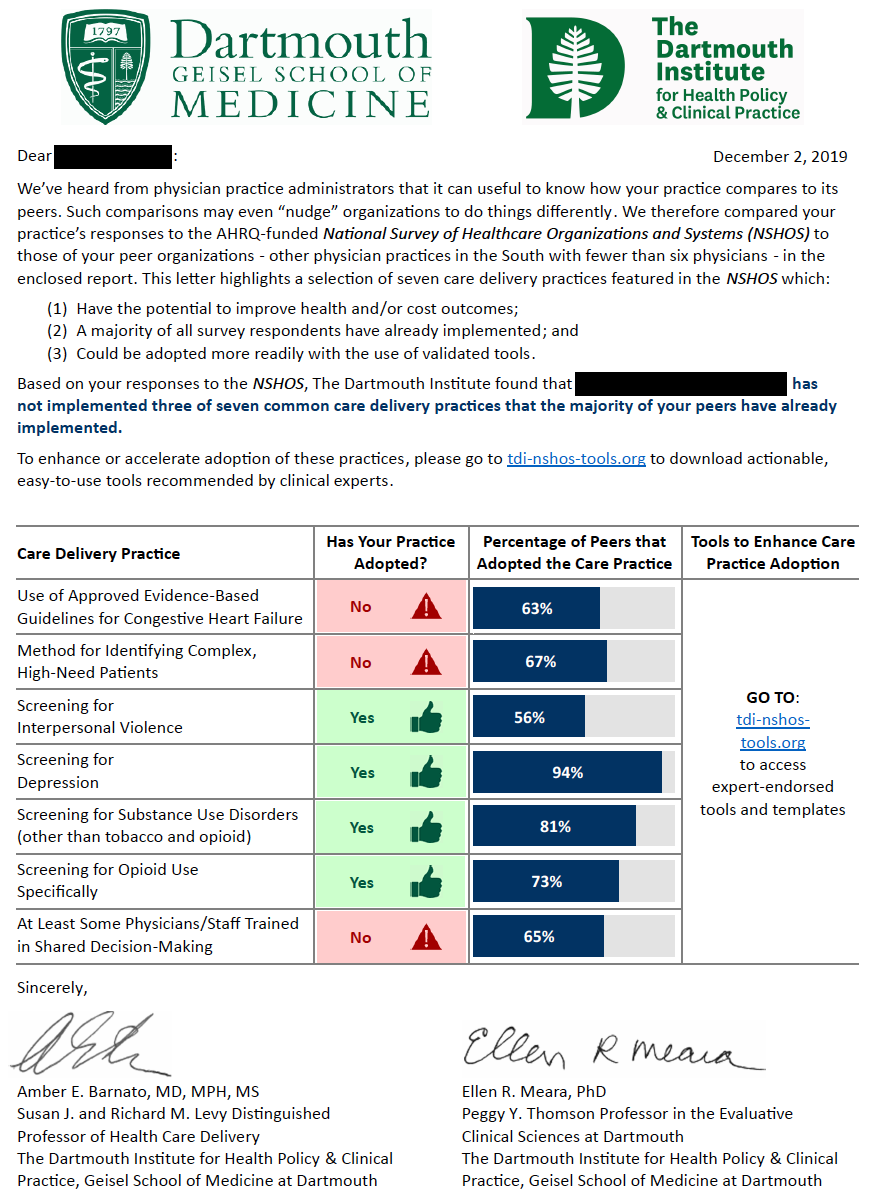

Supplement: S3 File — (DOCX) [file pone.0311442.s003.docx]

**S4 File. Control hard copy letter**


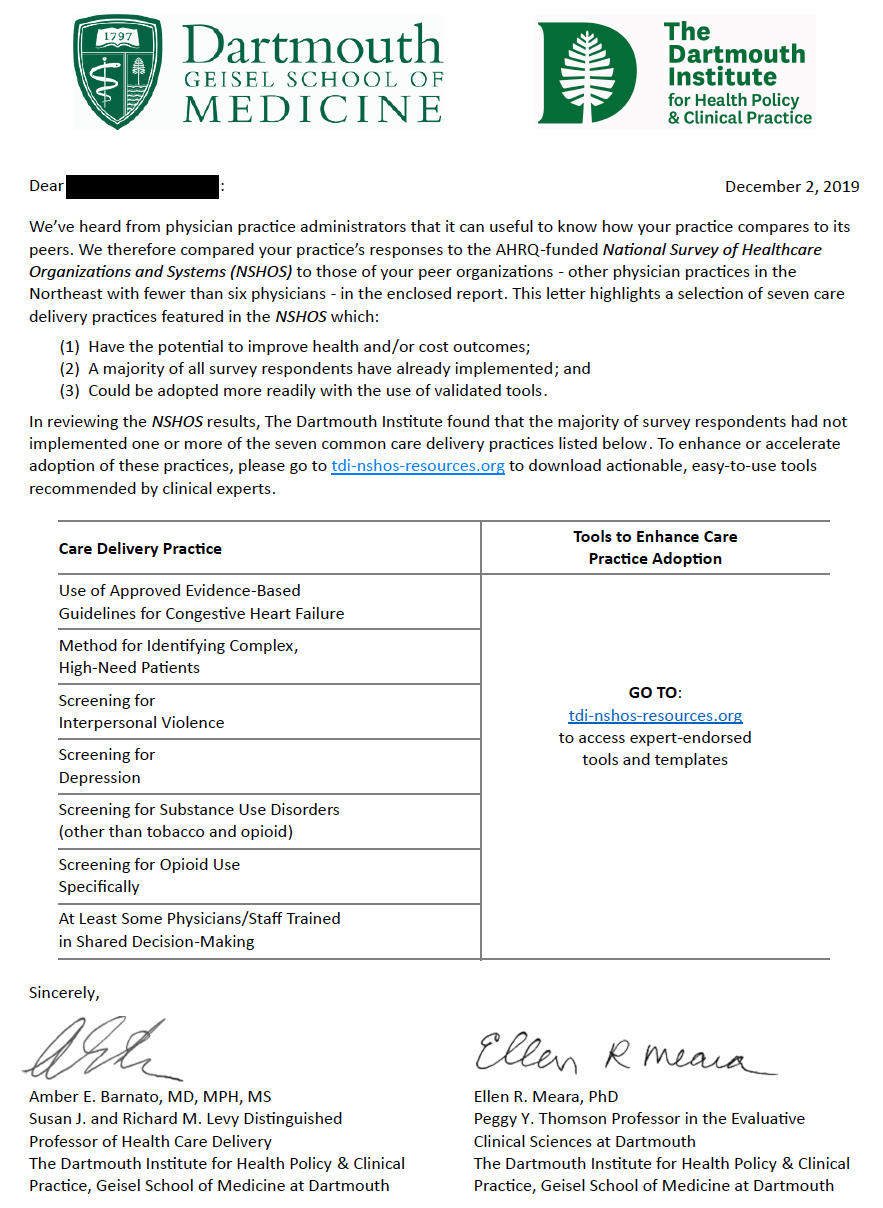

Supplement: S4 File — (DOCX) [file pone.0311442.s004.docx]

**S5 File. Resource website resource page screenshot**


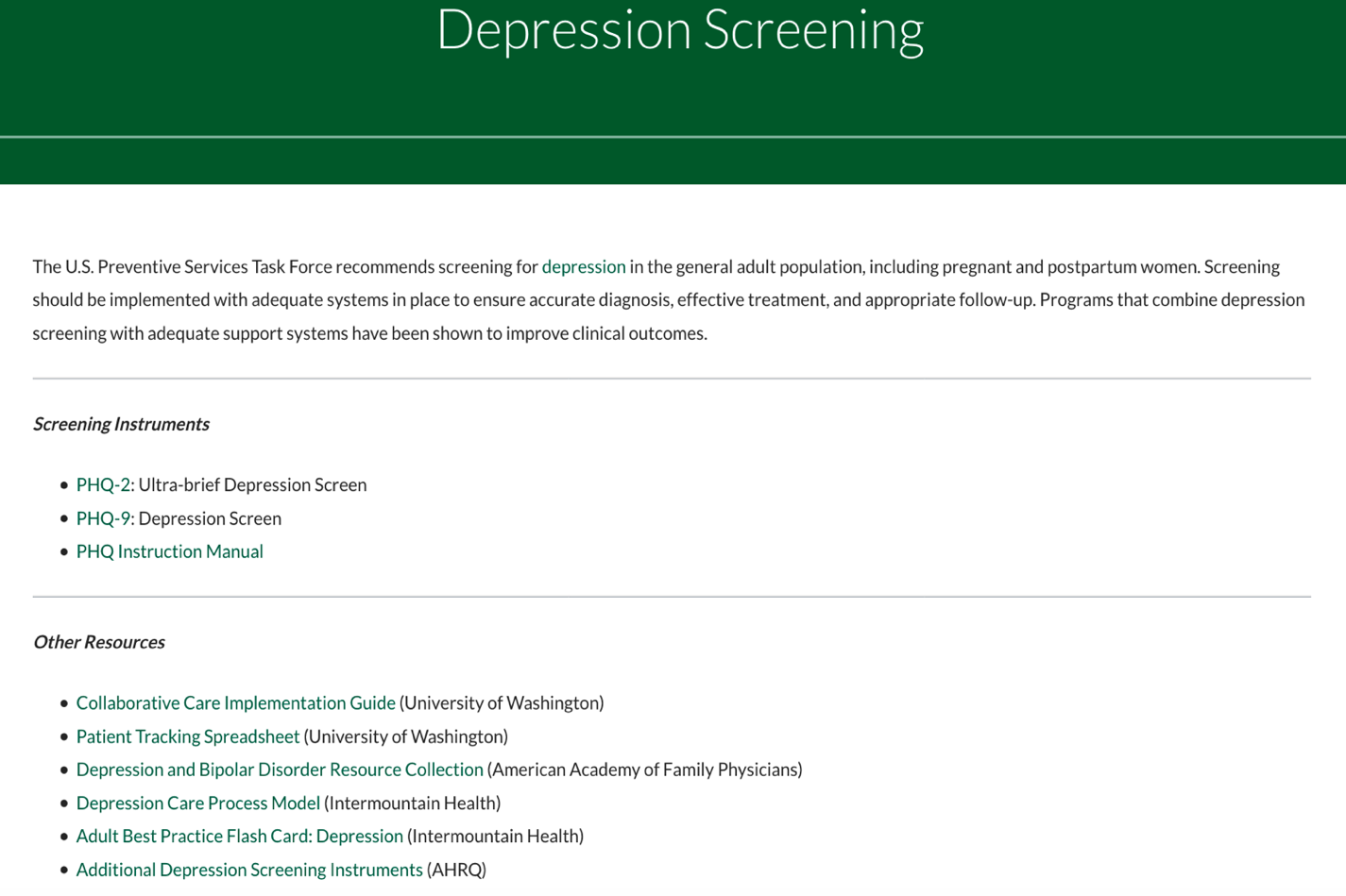

Supplement: S5 File — (DOCX) [file pone.0311442.s005.docx]
