# Supplementary material for: Assessing a behavioral nudge on healthcare leaders’ intentions to implement evidence-based practices
Source: PLoS One. 2024 Nov 22;19(11):e0311442. doi: 10.1371/journal.pone.0311442 (PMC11584086; doi:10.1371/journal.pone.0311442)
Supplement: S2 File — (DOCX) [file pone.0311442.s002.docx]

**S2 File. Supplementary Methods**

**NSHOS peer comparison reports**

The NSHOS is a set of three nationally representative surveys that collected data on the structure, leadership, and care delivery capabilities of physician practices, hospitals, and health systems. The NSHOS was in the field from June 16, 2017 – August 17, 2018. The peer comparison reports were developed to provide NSHOS respondents with aggregated, de-identified data on how they compared to their organizational peers on a subset of survey topics.

**Criteria for choosing care practices**

We used the following criteria to choose the target care practices: (1) the majority of total NSHOS respondents reported already adopting the practice (suggesting feasibility); (2) evidence suggests that the care practice can improve healthcare processes and/or patient health outcomes, per U.S. Preventive Services Task Force recommendations or clinical guidelines (strength of the evidence); and (3) publicly available tools exist to support or enhance organizational adoption of the care practice (more easily actionable). The seven care practices differed slightly between healthcare systems, hospitals and physician practices, based on the NSHOS survey instrument; additionally, a care practice was excluded for a particular peer group if less than 50% of the organizations in that group reported adopting the practice.

**Development of peer comparison groups**

To create peer groups, we classified physician practices into sixteen groups based on the number of physicians in the practice (1-5; 6-10, 11-20 and 20+) and the practice’s location (Midwest, West, South, and Northeast); hospitals into six groups (academic medical centers; critical access hospitals; and general acute care hospitals in the Midwest, West, South and Northeast); and healthcare systems into three groups based on organizational structure (medical groups, simple integrated systems, and complex integrated systems). See below for breakdown of the groups and the care practices included in each group’s peer comparison report.

| **Evidence-based Care Practices Featured in Cover Letter** | **Survey Level** | | |
| --- | --- | --- | --- |
|  | **Healthcare systems** | **Hospitals** | **Physician Practices** |
| Screening for opioid use | X | X | X |
| Screening for substance use disorders | X | X | X |
| Screening for depression | X | X | X |
| Screening for interpersonal violence | X^a^ | X | X^b^ |
| Method for Identifying complex high need patients | X^c^ | X | X |
| Evidence-based guidelines for congestive heart failure | X | X | X |
| Evidence-based guidelines for sepsis | X^d^ |  |  |
| At least some staff trained in shared decision-making |  | X | X |
| ^a^ Was not included in report for medical groups because less than 50% of medical groups reported adopting this practice  ^b^ Was not included in reports for the following physician practice classifications because less than 50% of these groups reported adopting this practice: practices with 20+ physicians in the Northeast; practices with 11-20 physicians in the South; practices with 6-10 physicians in the West; and practices with 11-20 physicians in the West.  ^c^ Was not included in report for medical groups because less than 50% of medical groups reported adopting this practice  ^d^ Was not included in report for simple integrated systems because less than 50% of simple integrated systems reported adopting this practice | | | |

**Randomization strata**

Randomization was performed with strata defined by: (1) general organization type (healthcare system, hospital, practice); (2) sub-organization type (medical group, simple system or complex system for healthcare systems; academic medical center, critical access hospital, or other acute care hospital for hospitals; small, medium, or large for physicians practices); (3) number of non-implemented care delivery practices from the starting list of up to seven (1, 2, 3, and 4+); and (4) with/without a listed email address.

**Power calculation**

With a sample of 2,387, the test statistic for the difference in proportions under the null hypothesis is very closely approximated by a normal distribution. Therefore, the observed value of the *Z*-statistic was compared to the 0.975 quantile of the standard-normal distribution, 1.96, to determine whether a statistically significant difference was obtained at the 0.05-level (a test statistic of magnitude 1.96 or greater would represent statistical significance).
