# Supplementary material for: Assessing a behavioral nudge on healthcare leaders’ intentions to implement evidence-based practices
Source: PLoS One. 2024 Nov 22;19(11):e0311442. doi: 10.1371/journal.pone.0311442 (PMC11584086; doi:10.1371/journal.pone.0311442)
Supplement: S6 File — (DOCX) [file pone.0311442.s006.docx]

**S6 File. Nudge email**

Dear [Name],

We are following up on the letter we recently sent you via U.S. Mail with your hospital's results compared to other acute care hospitals in the Midwest on the AHRQ-funded National Survey of Healthcare Organizations and Systems (NSHOS).

Based on your survey responses, The Dartmouth Institute found that **[organization] had not implemented two of seven common care delivery practices that a majority of your peer hospitals had already implemented**.

Click on the link below and enter your unique organizational passcode to see how your hospital measures up against your peers on these seven commonly employed care delivery practices with a strong evidence base. Following this comparison is your full NSHOS respondent report, which includes your organization's NSHOS responses and aggregated peer results on a range of topics.

**Report Portal: www.tdi-nshos.org 
Passcode: XXXXX**

**Access expert-recommended tools**: To support [organization]’s adoption of the care delivery practices it has not yet implemented, experts at Dartmouth have compiled a set of practical tools and resources meant to jump-start your implementation efforts.

Please click [here](https://tdi-nshos-tools.org/) to access this set of expert-recommended resources, as well as to sign up for the opportunity to connect with other hospitals about implementation best practices.

Sincerely,

[Name]

*Note: Clicking on the Report Portal link and entering the passcode enabled participants to view a pdf version of their personalized hard copy letter (Appendix 3) and the survey peer comparison report (Appendix 1)*
