# Supplementary material for: Assessing a behavioral nudge on healthcare leaders’ intentions to implement evidence-based practices
Source: PLoS One. 2024 Nov 22;19(11):e0311442. doi: 10.1371/journal.pone.0311442 (PMC11584086; doi:10.1371/journal.pone.0311442)
Supplement: S7 File — (DOCX) [file pone.0311442.s007.docx]

**S7 File. Control email**

|  |
| --- |

Dear [Name],

We are following up on the letter we recently sent you via U.S. Mail with your healthcare system's results compared to other similarly structured healthcare systems on the AHRQ-funded National Survey of Healthcare Organizations and Systems (NSHOS).

Click on the link below and enter your unique organizational passcode to see your full NSHOS respondent report, which includes your healthcare system's NSHOS responses and aggregated peer results on a range of topics.

**Report Portal: www.tdi-nshos.org 
Passcode: XXXXX**

**Access expert-recommended tools**: To support [organization]’s new or enhanced adoption of seven of the care delivery practices featured in the NSHOS, experts at Dartmouth have compiled a set of practical tools and resources meant to jump-start your implementation efforts.

Please click [here](https://tdi-nshos-resources.org/) to access this set of expert-recommended resources, as well as to sign up for the opportunity to connect with other healthcare systems about implementation best practices.

Sincerely,

[Name]

|  |
| --- |

*Note: Clicking on the Report Portal link and entering the passcode enabled participants to view a pdf version of their personalized hard copy (Appendix 3) and the survey peer comparison report (Appendix 1)*
