# Supplementary material for: Assessing a behavioral nudge on healthcare leaders’ intentions to implement evidence-based practices
Source: PLoS One. 2024 Nov 22;19(11):e0311442. doi: 10.1371/journal.pone.0311442 (PMC11584086; doi:10.1371/journal.pone.0311442)
Supplement: S8 File — (DOCX) [file pone.0311442.s008.docx]

**S8 File. Qualitative interview guide**

***Introduction script***:

Thank you for speaking with me today. My name is [NAME] and I work at [Organization Name] as part of a research team studying how messaging strategies impact the likelihood that health care administrators access resources to support the adoption of evidence-based care delivery practices. We are speaking with individuals at up to 30 organizations. Our conversation today should last less than 30 minutes and your participation in this interview is voluntary. I will be asking questions about a letter and personalized survey report that was sent to your organization in December 2019 and January 2020.

I would like to record our conversation, which may then be transcribed. Audio recordings and transcriptions will not include your name and will be stored in a password protected location. All information from this interview will be kept confidential, and any information used from this interview will be de-identified and cannot be linked back to you or your organization. We will keep audio recordings for three years after the conclusion of the study. During our conversation, you can skip questions as needed.

We are conducting this study for academic research purposes and plan to write academic papers detailing our findings. Do you have any questions before we begin?

Is it okay if I turn on my recorder now?

*(If yes, start recording.)*

Thank you.

***Background on NSHOS*:**

- We have a grant from the Agency for Healthcare Research and Quality to study health system performance and health care org’s use of evidence-based practices.
- As part of this work, we developed and fielded a national survey in 2017-18 to characterize the structure, ownership, leadership, and care delivery capabilities of health care systems, physician practices, and hospitals. We received responses from about 3,400 organizations – including yours.
- When we sent out the initial survey, we indicated that we’d send all respondents a report to summarize the survey results – which we mailed and emailed to you in December of last year. The report included a cover letter with some messaging and graphics regarding your organization’s reported adoption of 7 evidence-based care practices included in the survey.
- The purpose of our call today is to learn about your reactions to this cover letter, so we can understand how messaging and information framing strategies impact the perceptions and behaviors of health care leaders.

***Interview Questions****:*

- Recollection of filling out NSHOS survey in 2017-2018 (and ability to connect the report to the survey)?
- Recollection of receiving email and interest in opening it (and paper version)?
- What was your initial reaction when you saw the cover letter? What were you thinking/feeling upon looking at it? Any immediate emotions?
- Does it matter to you how you compare to your peers on the adoption of evidence-based care practices? Do you find this to be a powerful motivator?
- Did you click on the link in the cover letter – or in the email itself – to access a webpage with technical assistance resources?
  - Why/why not?
- *IF YES:*
  - *General perception of/reaction to technical assistance website*
  - *Resource pages viewed and the utility/helpfulness of the available resources*
  - *Choice to download/not download resources*
    - *If downloaded, intention/likelihood of using*
  - *Thoughts on more effective resources or support*
  - *Interest in engaging with other organizations about strategies to promote adoption of evidence-based care delivery practices*
- Any immediate actions taken following the viewing of letter and report (e.g., Forwarding, reaching out to a colleague, etc.)?
- Potential future actions spurred by viewing letter and report related to adoption of evidence-based care practices? Does this change your organization’s actions or priorities?
- Other motivators for working to adopt new evidence-based care practices?
