# Supplementary material for: Assessing a behavioral nudge on healthcare leaders’ intentions to implement evidence-based practices
Source: PLoS One. 2024 Nov 22;19(11):e0311442. doi: 10.1371/journal.pone.0311442 (PMC11584086; doi:10.1371/journal.pone.0311442)
Supplement: Attachment — Submitted filename: Response_to_Reviewers_Nudge.docx [file pone.0311442.s009.docx]

Alberto Molina Pérez, Ph.D.
Academic Editor
*PLOS ONE*

February 29, 2024

Dear Dr. Pérez,

On behalf of my co-authors, I would like to thank you for the opportunity to revise our manuscript entitled “Assessing a behavioral nudge on healthcare leaders’ intentions to implement evidence-based practices” for your consideration in *PLOS ONE*. We have revised the manuscript to respond to the peer reviewers’ thoughtful comments, and we are grateful for this opportunity to improve our work. To this end, we are very open to any further edits that you suggest. We have included reviewer comments below, along with our responses and revisions. Manuscript revisions made in response to reviewer comments are identified with red and bold text.

We appreciate your consideration of our manuscript.

Sincerely,

Maia Crawford (corresponding author)

**Reviewer Comments**

This study examined whether a letter with social norms messaging and peer comparison data impacted healthcare leaders’ decision to access a website with resources to support
evidence-based practice adoption. The team conducted a pragmatic, randomized controlled trial of 2,387 healthcare leaders from health systems, hospitals, and physician practices in the United States to receive one of two cover letter versions, each linking to a resource website and accompanying a report with survey results. The “nudge” letter included messaging that highlighted how an organization’s results compared to peers using text, color, and icons. The letters were sent in Dec 2019 and emails and followup emails were sent Dec 2019 and Jan 2020 to those with email addresses. The team interviewed 14 participants to understand how the letter and report impacted behaviors. 1.8% of the leaders sent the nudge letter accessed online resources, compared to 1.4% sent the control letter (p=0.424).

Overall, this study makes an excellent contribution to several areas of the literature in terms of applied behavioral science and nudge interventions in health care, health care quality improvement, and implementation science. The nudge literature has been criticized for publication bias. It’s critical that rigorous randomized trials with null findings like this are published to better understand what works and what doesn’t. The nudge interventions were well conceived and it’s likely that many would have thought they could be effective at increasing engagement with quality improvement interventions. The results will be informative to quality improvement leaders as they plan future interventions to increase adoption of evidence-based practices.

I have some recommendations for improving the paper and it’s impact:

1. More clarity needed in description of how the intervention was delivered.
a. I had to read through the methods and the supplemented several times to understand how the intervention was delivered. It should be stated more clearly that the letters were sent by postal mail to all individuals who were randomized and that an email was sent to ~60% of the randomized individuals who had an email address. S1 Figure 1 makes this much more clear than Figure 1. S1 Figure 1 should be included in the main manuscript instead of the current Figure 1.

**Response:** We agree that more clarity was needed regarding how the intervention was delivered, and have therefore added in information about sending the letters via both postal mail and email to the abstract, added in the percentage of participants who received a letter via email, and moved the paragraph about the mode of letter delivery earlier in the Methods section:

Abstract, page 2: We randomized 2,387 healthcare leaders from health systems, hospitals, and physician practices in the United States in a 1:1 allocation ratio to receive one of two cover letter versions **via postal mail (all) and email (for the 60.6% with an email address)**…

Methods section, page 9:

**Intervention Delivery**

**Hard-copy** **cover** letters and survey reports were mailed via the U.S. Postal Service to all 2,387 study participants between December 6 and December 10, 2019. Cover letters and survey reports were **additionally** emailed to all organizations with an email address on file on December 18, 2019 (n=1,446**, 60.6% of study participants**). **E-mail recipients had to click on a hyperlink and enter a unique, five-character password contained in the same e-mail to access a downloadable pdf of their personalized letter and report. We then sent** two additional emails to individuals who had not opened the previous email(s) on January 8 and January 20, 2020 (S6-7 Files, S1 Fig).

We have also replaced Figure 1 with Figure S1.

b. Given that over 86% of those who were emailed the letter didn’t open the email, it’s critical to know what the subject line of the email was. This should be stated in the manuscript and included in the supplement.

**Response:** Thank you for this excellent suggestion; we now include this information in the manuscript and supplement.

Methods section, page 9: **The subject line for the first email was: “See how you compare: Your AHRQ-funded survey results.” Subject lines for the subsequent reminder emails were: “Thank you for completing the AHRQ-funded survey; See your results” and “AHRQ-funded survey results just released; see how you compare.” We did not pilot test e-mail subject lines.**

c. It should also be more clearly stated that the email required a login step to get into the report

**Response:** We appreciate this suggestion and agree. We have updated the manuscript with this information.

Methods section, pages 5: …**we emailed all NSHOS respondents for whom we had an email address, and provided them with a hyperlink to access their personalized cover letter and report using a passcode contained in the same e-mail.**

Methods section, page 9**:** … **E-mail recipients had to click on a hyperlink and enter a unique, five-character password contained in the same e-mail to access a downloadable pdf of their personalized letter and report.**

2. Calling out important exploratory outcomes in the Results. This is mentioned in the discussion, but not called out in the Results.
a. The fact that unique website resources downloads was no different, and likely even more common in the control group is something that should be called out because that’s the final behavior the researchers are trying to improve. This is less that 2% overall of intended recipients, indicating that the efforts were unsuccessful for 98% of the targeted recipients.

**Response:** We have added a subsection on secondary outcomes to the Results section, which describes the number of unique website resource downloads across groups:

Results section, page 13:

**Secondary Outcomes**

**As summarized in Exhibit 2, a total of 49 resources were viewed or downloaded; 16 were viewed on the nudge website and 33 on the control website (21 of the 33 control site downloads resulted from one visitor). Two website visitors requested a connection to a peer organization (an option available on the resources webpages): one nudge website visitor and one control website visitor.**

3. Discussion and key lessons need to be reframed around the major findings of the trial.
a. Last line of the discussion 266-267 – “The qualitative results, however, suggest
that providing administrative leaders with data comparing their organization’s performance to that of peers can spur follow-up action aimed at promoting evidence-based practice adoption” needs to be reframed. These qualitative results were only collected among 2% of the intended recipients who engaged with the intervention. Would reframe the qualitative findings to be among the small proportion of the intended recipients who engaged with the intervention. Furthermore, the qualitative methods are limited and don’t include a conceptual model and with very little description of how the responses were coded and analyzed. I would worry that the quotes were cherry picked to add some positive findings around the intervention. I’m not sure that very much can be concluded from the qualitative analysis since engagement was so low and engagement is likely reflective of highly motivated individuals. This is even stated as a limitation at the end that “ the qualitative interviews were conducted with a small sample, so it is hard to draw broad-based conclusions from them.”

**Response:** We appreciate this comment and have reframed how we discuss our qualitative findings in the Discussion section.

Discussion section, page 18: **While our qualitative data likely reflect the perceptions of particularly motivated and engaged leaders, the fact that nine of 14 interviewed leaders shared the materials with colleagues or initiated other subsequent activities suggests the peer comparison reports themselves provided a baseline incentive to act, and that the social norms messaging in the nudge letter did not provide an additional marginal incentive.**

Discussion section, page 19**:** Fifth, the qualitative interviews were conducted with a small sample **of leaders who viewed their reports and agreed to be interviewed, suggesting a high degree of interest and engagement in the study topic but making it** hard to draw broad-based conclusions **applicable to all healthcare leaders** from **the qualitative results.** 

b. The stated three lessons from the results do not align closely with the key findings of the trial, particularly the first two: “1) peer comparison data can be powerful…and 2) Outcome variables should align.” The key finding of the study is that a letter distributed through postal mail and email was that 86% of health care leaders didn’t open the email, and only 1.4% of the leaders visited the website with implementation resources. The other key finding was that even among the few who engaged, there wasn’t a significant difference in engagement according to whether the letter was reframed with insights from behavioral science. This suggests that biggest opportunity is around how to engage and capture the attention of health care leaders. This is a much bigger opportunity that trying to optimize messaging once engaged.

**Response:** Thank you for this helpful perspective on the key lessons to draw from the paper. We have decided to rewrite the Discussion section using a more standard approach of reviewing the findings in the context of the broader body of research and drawing implications, rather than calling attention to three specific lessons. We agree that more emphasis should be placed on the need to engage and capture the attention of healthcare leaders and have added this into the Discussion section and Conclusion. We specifically added in a subsection noting the implications of our findings, where we highlight the importance of using strategies to help maximize the number of study participants who open emails and view study materials.

Discussion section, pages 20-21:

**Implications**

**As we were unable to test the impact of our cover letters among a large group (only 89 of over 2,300 study subjects electronically viewed their letters and had known access to the website link), future studies targeting healthcare leaders should invest more time and resources in optimizing communication materials’ design and dissemination. Opportunities to enhance email-based studies include using a more attention-grabbing email sender – such as a known local contact or a national authority figure – and obtaining more up-to-date and high-quality contact information. Additionally, researchers should personalize subject lines and be purposeful about scheduling email deliveries at times associated with higher open rates.**

c. Much more emphasis of the discussion should be focused on what is known re engagement/open rates of letters/emails for engaging health care leaders and clinicians. The findings should be placed in this context and opportunities to optimize in the future including the messenger, subject line, and steps to decrease friction to reaching web resources (getting rid of login steps).

**Response:** We now spend more time in the Discussion section on issues related to why more study subjects did not open their open their emails and how promote higher open rates in future studies.

Discussion section, page 19: **The intervention’s low impact could also relate to weaknesses in how the letter was designed and delivered. First,** many leaders never received or opened their letter and report**.** While we do not know the open rate for the paper letters **– an inherent shortcoming of that delivery modality – the 13.7% email open rate is at the low end of the 9.3% – 46.0% range for studies in which physicians were emailed to take an online survey or view web-based materials (we are unaware of comparable data for healthcare executives or similar business leaders). Our study’s low open rate suggests that the email’s subject line, which was not personalized,** did not capture the attention of busy, high-ranking healthcare leaders who receive hundreds of emails per day**; the emails may also have been dismissed as spam or marketing. Among those who opened the email, about half accessed their reports. Removing the additional passcode entry step would likely further increase report download rates. Finally, we sent the letters in December 2019 and January 2020, a time when leaders may have been busy with end-of-year activities, budget planning, vacations, and early preparations for the COVID pandemic.**

d. I suspect that one factor associated with low engagement in Dec 2019 and Jan 2020 among health care leaders was that there was a deluge of email communications and meetings occurring around that time with regards to planning for the Covid-19 preparedness and also December and January are difficult times to engage health care leaders since this is the beginning of budget planning season and there many high priority meetings stacked just before and after the holidays. It may be worth repeating at other times of year outside of the emergence of a new pandemic.

**Response:** This is a great point; while we considered the impact of pandemic-related responsibilities and competing priorities on the low participation rate for the qualitative interviews, we had not considered the impact of the December 2019 - January 2020 timeframe on the email open rate. We have now added this as a consideration in the Discussion section:

Discussion section, page 19: **Finally, we sent the letters in December 2019 and January 2020, a time when leaders may have been busy with end-of-year activities, budget planning, vacations, and early preparations for the COVID pandemic.**

e. It seems like a major missed opportunity here was a lack used-centered design and feedback from health care leaders and pilot testing of alternative nudge strategies before full deployment. Little details of design can have outsized impacts. This should be highlighted as opportunity for future optimization.

**Response:** The team pilot tested our study approach and draft materials prior to the study. We have now included a description of these efforts in the manuscript. We agree, however, that more feedback and focus on user-centered design should be highlighted as a key opportunity for future studies and have thus also noted this in the Discussion section.

Methods section, page 7: **To develop the intervention (or nudge) cover letter, the study team pilot tested draft materials and dissemination strategies with two high-level health system executives, one health services researcher with expertise in behavioral nudges, and a Dartmouth research team that develops and tests patient and provider decision support tools, iteratively modifying materials after each round of input. We also received feedback on materials to include on the resource webpages from 10 clinical subject matter experts.**

Discussion section, page 21: **The study team should also invest in more intensive pilot testing, employing an iterative, user-centered design approach of eliciting feedback and revising materials through successive rounds of testing.**

Reviewer #2: The study explores the efficacy of a behavioral nudge in influencing healthcare leaders.

A total of 2387 healthcare leaders in the United States were randomized to receive one of two types of cover letters. Both letters provided a link to a resource website and a report with survey results, but the "nudge" letter included additional elements like text color and icons to highlight how an organization’s results compared to the peers.

There were no statistically significant effects of the intervention on website access, and regardless of cover letter format, website access was low, with less than 2% of all organizational leaders targeted, clicking through to website resources.

According to the authors, the qualitative results suggest, however, that providing administrative leaders with data comparing their organization’s performance to that of peers can spur follow-up action aimed at promoting evidence-based practice adoption.

Study Design and Methodology:

The pragmatic parallel-group randomized controlled trial approach is appropriate for the study's objectives. However, the manuscript would benefit from elaborating on the selection criteria for these leaders.

**Response:** Thank you for this suggestion; we have added in additional information in the Methods section regarding selection criteria for the leaders in our study, who were leaders who previously filled out the National Survey of Healthcare Organizations and Systems (NSHOS). We elaborate on how these leaders were initially chosen to participate in the NSHOS:

Methods section, pages 6: **This study relied exclusively on the NSHOS study sample and associated contact information. The NSHOS sample frame was developed using data from the 2015 IQVIA OneKey database, which included organizations’ physical and postal addresses, along with supplemental data from the American Hospital Association and AHRQ. Once organizations were sampled, research assistants worked to verify the data provided and gather names, email addresses, and phone numbers of organizational leadership.**

**Chief Executive Officers, Chief Medical Officers, and Chief Clinical Officers were targeted to** fill out the NSHOS from health systems and hospitals**, with health system Chief Administrative Officers and Chief Clinical Officers and hospital Chiefs of Medicine and Medical Staff serving as secondary targets. Practice managers, administrators, or lead physicians were targeted to fill** out the survey at physician practices**; secondary targets were currently practicing primary care physicians.**

The distinction between the "nudge" letter and the control letter should be more pronounced in the manuscript. A clearer explanation of the specific elements used in the nudge letter (e.g., text color, icons) and -especially - how they are expected to influence behavior, would be beneficial.

**Response:** We agree with this suggestion and have added more details about how the two cover letters differed in the manuscript.

Methods section, page 7: **Following a short introduction, the nudge letter stated: “[organization name] has not implemented [x] of seven common care delivery practices that the majority of your peers have already implemented.” This statement used descriptive norms to directly compare the leader’s organization to its peers, a strategy meant to demonstrate that evidence-based care practice adoption was the norm among similar organizations. It then included a table listing the seven evidence-based practices and indicating whether the leader’s organization adopted each practice using a green thumbs up for adoption and a red caution triangle for non-adoption. The use of color coding and positive and negative icons employed injunctive norms – the inference of others’ approval – to convey approval for adoption and disapproval for non-adoption (S3 File). The table also included peer comparison data for the seven featured care delivery practices, using horizontal bars to display the adoption percentage for each care practice among peer organizations. To the right of the peer comparison data was a hyperlink to access “tools to enhance care practice adoption.”**

The control letter **included the same introductory paragraph, but without the statement noting the organization’s number of non-adopted practices. Like the nudge letter, the control letter had a table listing the seven featured care practices, but this table did not include data about whether the organization adopted each practice (and therefore, no colors or icons), nor did it contain any peer comparison data. It only included the same hyperlink to online tools (S4 File).**

The manuscript should include a more comprehensive discussion on the implications of the relatively low response rate (1.8% for the nudge letter and 1.4% for the control letter).

**Response:** We appreciate and agree with this suggestion and have reframed the Discussion section to focus more on why the website access rate was so low and steps that researchers can take to improve these rates in future research, as described in sthe response to Reviewer 1 above.

Literature Review and Theoretical Framework:

The introduction provides a good foundation for the study, highlighting the challenges in integrating evidence-based practice in healthcare and the role of behavioral nudges. However, it could be strengthened by incorporating more recent literature to establish the current state of research in this field. This would position the study more firmly within the existing body of knowledge.

The manuscript should explore in greater depth the theoretical underpinnings of nudging in the context of healthcare leadership. Discussing theories of behavioral economics in relation to healthcare leadership decision-making could provide a richer theoretical framework.

**Response:** Thank you for this suggestion; in the Introduction nwe have added new citations for recent studies on behavioral nudges in healthcare, as well as expended on the theoretical underpinnings of nudging healthcare leaders. However, one impetus for the study was a lack of research (theoretical or empirical) regarding behavioral nudges of healthcare leaders.

Introduction, page 3: **Recent systematic reviews of clinician-directed nudges affirm that nudges can improve clinical decision-making and effectively promote adherence to evidence-based clinical and administrative guidelines.**

Introduction, page 4: **We sought to devise an experiment to test the efficacy of nudging individuals who serve as “decision architects” for their respective organizations, delivering a more “upstream” intervention to change clinical practice behavior not at the clinician level, but at the decisionmaker level. This work assumes that healthcare leaders maintain an “intention-behavior gap” related to evidence-based practice adoption: a discrepancy between what they plan or aspire to do and what they, by way of their organization, do in practice. A number of existing frameworks help explain why decisionmakers’ actions may not always align with their intentions, including the Theory of Planned Behavior, which describes how a person’s actions are related to their beliefs about the behavior’s consequences, others’ expectations, and inhibiting or enabling factors. Subsequent behavioral frameworks consider additional factors that influence behavior, like infrastructures, the political environment, and economics.**

Results and Discussion:

The discussion section is unsatisfactory at the moment. It needs to delve deeper into the implications of the results. Specifically, a more critical analysis of why the nudges did not significantly increase website access is needed.

**Response:** We agree this will strengthen the manuscript. As noted above in response to Reviewer 1, we have made significant edits to the Discussion section; it is now more focused on why website access was low, and what researchers can learn from this study to improve the design and implementation of future work.

The qualitative insights from the interviews are valuable but are not sufficiently integrated into the overall discussion. A more thorough analysis of these insights in relation to the quantitative findings would offer a more nuanced understanding of the study's implications.

**Response:** Thank you for this suggestion. We have added additional analyses of the qualitative insights and how they related to the quantitative fundings in the Discussion section:

Discussion section, page 18: **When the quantitative and qualitative results are interpreted together, we find that the nudge letter recipients did not take more or different actions than control letter recipients: a similar number of participants in both groups visited the website, forwarded the reports, and took other subsequent actions. While our qualitative data likely reflect the perceptions of particularly motivated and engaged leaders, the fact that nine of 14 interviewed leaders shared the materials with colleagues or initiated other subsequent activities suggests the peer comparison reports themselves provided a baseline incentive to act, and that the social norms messaging in the nudge letter may not have provided an additional marginal incentive.**

Technical Aspects and Presentation:

The manuscript generally follows a clear structure, but there are areas where the flow can be improved for better readability. For example, transitions between sections, particularly between the methodology and results sections, could be smoother.

**Response:** We have reorganized and retitled subsections of the methods to increase readability and improve transitions in the Methods section.

In summary, the manuscript presents an interesting study with potential implications for healthcare leadership and practice. However, to fully realize its contribution to the field, the above-mentioned revisions are necessary. The focus should be on strengthening the theoretical framework and deepening the analysis of the results (both quantitative and qualitative).

I would also suggest to extend the Conclusions section of the article.

**Response:** We appreciate your helpful feedback and have worked to strengthen the manuscript by incorporating the suggested edits. We have also expanded the Conclusion section:

Conclusion section, pages 21-22:

**This study tested whether healthcare leaders were more likely to access a resource website after viewing a cover letter with behavioral science-inspired messaging and data display. We did not find a statistically significant difference in website views between those who received the nudge and control letters. The low email open and report download rates for all study subjects imply shortcomings in the execution of our letter-based experiment that likely led to low website viewing rates.

The quantitative and qualitative results together suggest that actions taken following the viewing of the letters and reports were unlikely to be associated with the cover letter messaging, as the two study arms’ subsequent actions did not discernably differ. The fact that control letter recipients took any actions suggests that they may have been motivated by the data in their personalized reports, which compared survey responses to those from similar healthcare organizations. Future research that targets administrative healthcare leaders to improve organization-wide practice adoption should make purposeful design and dissemination choices; invest in user-centered pilot testing; and align interventions with leaders’ motivations, priorities, and workflows.**
