# Supplementary material for: Assessing a behavioral nudge on healthcare leaders’ intentions to implement evidence-based practices
Source: PLoS One. 2024 Nov 22;19(11):e0311442. doi: 10.1371/journal.pone.0311442 (PMC11584086; doi:10.1371/journal.pone.0311442)
Supplement: Attachment — Submitted filename: Response to Reviewer Comments 09_16_24.docx [file pone.0311442.s010.docx]

Laura Brunelli, MD, PhD
Academic Editor
PLOS ONE

September 16, 2024

Dear Dr. Brunelli,

I would like to thank you for the opportunity to revise our manuscript entitled “Assessing a behavioral nudge on healthcare leaders’ intentions to implement evidence-based practices” for your consideration in *PLOS ONE*. My coauthors and I have revised the manuscript to respond to the peer reviewers’ thoughtful comments, and we are grateful for this opportunity to improve our work. To this end, we are very open to any further edits that you suggest. We have included reviewer comments below, along with our responses and revisions. Manuscript changes made in response to reviewer comments are in italicized and bolded text.

We appreciate your consideration of our manuscript.

Sincerely,

Maia Crawford (corresponding author)

**Reviewer Comments**

Reviewer #1: The authors did a very nice job responding to the reviewer critiques and suggestions. This will be a nice contribution to the literature.

Reviewer #3: The authors report on a randomized control trial undertaken to test the effectiveness of focused communication strategies, informed by behavioural nudge theory, in influencing healthcare leaders to adopt evidence-based care delivery practices. The novel aspect of this study is the focus on healthcare leaders as opposed to clinicians given that these leaders often have the decision-making authority to promote and oversee the implementation of improvements. The methods were described in sufficient detail and were appropriate for the study aims. Ethics approval for the study was noted. The statistical analysis was appropriate given the limited data to be analyzed. The qualitative analysis of the transcribed interviews was explained briefly but with sufficient detail given the purpose of the study. The discussion flowed logically and considered important ways to understand the findings. The limitations were realistic and demonstrated important insights into the challenges experienced in conducting the study. Despite the very low response rate for the quantitative measure to assess the intervention outcome (visiting the website resources) and the non-significant statistical results, the report offers many insights into the issue of how organizations adopt evidence-based change and likewise how to study the impact of strategies to promote adoption through the use of nudge theory. Overall, the study report is well written and was a pleasure to read.

While there are no critical gaps in the report, a few suggestions are offered for consideration only. They reflect my limited knowledge of nudge theory and the desire to understand some aspects of the study in more detail. Some minor edits are also mentioned.

Introduction – It might be useful to add more detail about nudge theory to briefly explain the mechanism by which people (i.e., leaders) are influenced. As I read the paper, I also wondered how nudge-based strategies fit (or not) into implementation science concepts.

***Response****: We appreciate this suggestion and agree that additional context about the mechanisms by which nudges influence individuals would be helpful to include in the Introduction. We have therefore added in a few sentences to better describe this process, as well as how nudge-based strategies fit into the larger field of implementation science:*

*Introduction, Page 4, Lines 110 - 116: “****Nudges can help those interested in implementing evidence-based practices overcome organizational constraints. They do this by altering aspects of one’s decision-making environment (known as “choice architecture”)(******6******) to make implementation feel easier or more salient; this can be done by changing the placement or attributes of objects or modifying messaging.(******5******,*** ***29******,*** ***30******) Nudging is therefore one strategy among many to promote or strengthen implementation science efforts, which encourage the uptake of evidence-based practices to improve the quality and efficacy of health services.(******31******,*** ***32******)****”*

Table 2 – I was confused by the use of the notations ‘a’ and the asterisk as the explanation at the bottom of the table does not differentiate ‘a’ from ‘*’. As it is currently stated, only an asterisk is needed.

***Response****: The “a” referenced above appears to be a formatting glitch that appeared for the reviewer. An “a” does not appear in our version of Table 2; we currently only include asterisks and no letter-based superscripts.*

Table 3 – The qualitative themes and exemplars are described clearly. I was left wondering if there were any comments about the nature or quality of the nudge messaging specifically. Most the themes were about actions but not the qualities of the actual intervention (social norm messaging and comparison charts).

***Response****: Thank you for this suggestion. Yes, we did ask interviewees who received a nudge letter about their perceptions of the nudge messaging and data display. We have now added in additional comments that reflect these reactions:*

*Table 3, Page 15:*

*Specific Findings:* ***“Found the nudge messaging and peer comparison data impactful or persuasive.” (nudge letter recipients) (nudge=6/8)***

*Exemplar Quotes:* ***“Seeing a "no" response when peers had a "yes" was a motivator” (Physician practice leader, nudge letter).***

***“My eyes immediately went to the thumbs up and the red triangle.” (Hospital leader, nudge letter).***

Discussion – It would be useful to link back the original aim and mention nudge theory or strategies. The word ‘nudge’ only appears twice on page 15 but nowhere else in the discussion section onward. Since nudge theory played an important role in the design of the intervention (and it is in the report title), it would be useful to explicitly state what has been learned about nudge-based strategies and this field of study.

***Response****: We appreciate and agree with this suggestion and have worked to better link the Discussion section back to nudge theory, which we described in the Introduction:*

*Discussion, Page 19, Lines 375 – 381:* ***“The fact that our study subjects – data-driven, outcomes-oriented, business-minded individuals – appeared more influenced by the peer comparison data demonstrates the importance of accounting for audience characteristics and situational context when applying nudge theory and designing nudge strategies. In retrospect, use of peer comparison data seems highly appropriate for leaders who need to make reflective professional decisions; social norms messaging and iconography – which produce automatic, almost subconscious reactions – may be more effective nudge strategies for individuals who need to make quicker, simpler decisions.”***

Figure 1 – The final box under each arm of the randomized groups (bottom of diagram) seems redundant i.e. ‘allocated to control letter’ and ‘included in the analysis’ are the same numbers. The diagram could logically end with the box for ‘received allocated intervention’.

***Response****: In the initial set of comments, a reviewer suggested that we replace Figure 1 with Figure S1, which provided a more clear and straightforward visualization of the study steps. We agreed with this suggestion and made the replacement. The current reviewer is referencing the original Figure 1, which we thought we had removed in the last round of edits. We apologize if we inadvertently uploaded the wrong version of Figure 1. We will be sure to upload the correct version, which does not have any redundancies and no longer has a box labeled “included in the analysis.”*
